# Insulin-Mediated Changes in Tau Hyperphosphorylation and Autophagy in a *Drosophila* Model of Tauopathy and Neuroblastoma Cells

**DOI:** 10.3389/fnins.2019.00801

**Published:** 2019-08-02

**Authors:** Shreyasi Chatterjee, Suren. S. Ambegaokar, George R. Jackson, Amritpal Mudher

**Affiliations:** ^1^Department of Neurology, Mitchell Center for Neurodegenerative Diseases, The University of Texas Medical Branch at Galveston, Galveston, TX, United States; ^2^Department of Biological Sciences, University of Southampton, Southampton, United Kingdom; ^3^Department of Botany and Microbiology, Ohio Wesleyan University, Delaware, OH, United States; ^4^Department of Neurology, Michael E. DeBakey VA Medical Center, Parkinson’s Disease Research Education and Clinical Center, Baylor College of Medicine, Houston, TX, United States

**Keywords:** Alzheimer’s disease, type 2 diabetes, tau aggregation, autophagy, tau hyper-phosphorylation

## Abstract

Almost 50 million people in the world are affected by dementia; the most prevalent form of which is Alzheimer’s disease (AD). Although aging is considered to be the main risk factor for AD, growing evidence from epidemiological studies suggests that type 2 diabetes mellitus (T2DM) increases the risk of dementia including AD. Defective brain insulin signaling has been suggested as an early event in AD and other tauopathies but the mechanisms that link these diseases are largely unknown. Tau hyperphosphorylation is a hallmark of neurofibrillary pathology and insulin resistance increases the number of neuritic plaques particularly in AD. Utilizing a combination of our *Drosophila* models of tauopathy (expressing the 2N4R-Tau) and neuroblastoma cells, we have attempted to decipher the pathways downstream of the insulin signaling cascade that lead to tau hyperphosphorylation, aggregation and autophagic defects. Using cell-based, genetic, and biochemical approaches we have demonstrated that tau phosphorylation at AT8 and PHF1 residues is enhanced in an insulin-resistant environment. We also show that insulin-induced changes in total and phospho-tau are mediated by the crosstalk of AKT, glycogen synthase kinase-3β, and extracellular regulating kinase located downstream of the insulin receptor pathway. Finally, we demonstrate a significant change in the levels of the key proteins in the mammalian target of rapamycin/autophagy pathway, implying an increased impairment of aggregated protein clearance in our transgenic *Drosophila* models and cultured neuroblastoma cells.

## Introduction

Alzheimer’s disease is a widely prevalent form of dementia that is characterized by loss of memory and other cognitive functions required to perform complex daily activities (Citron, 2004). Aging remains the most important risk factor for AD, but population-based studies have identified T2DM, an age-associated chronic metabolic disorder, as a major risk factor for developing cognitive impairment and dementia (Barbagallo and Dominguez, 2014; Sridhar et al., 2015). Insulin resistance in peripheral tissues is a key feature of T2DM, and accumulating evidence suggests that insulin resistance also develops in AD and other tauopathies in early stages of the disease (Goncalves et al., 2019). Recent studies have also shown that brain insulin resistance can develop independently from systemic insulin resistance. However, the underlying mechanisms that lead to persistent brain insulin resistance and needs to be investigated (Triani et al., 2018). Several studies have demonstrated that both insulin and insulin receptors (IRs) are found in the brain, and IR are highly expressed in neurons of the central nervous system (CNS) (Talbot et al., 2012; O’Neill, 2013). FDG-PET studies in AD patients have shown a progressive impairment of cerebral glucose uptake and metabolism particularly in the parieto-temporal lobes and posterior cingulate cortical regions of the brain that correlates strongly with disease progression (Moloney et al., 2010). Moreover, insulin and insulin-sensitizing drugs improve cognitive performance in people at early stages of AD (Freiherr et al., 2013; Roberts et al., 2014). The physiological and pathological role of IR signaling in the CNS is still unknown, but a strong correlation between AD and dysfunction of the insulin-signaling pathway with regard to glucose metabolism in the brain has prompted some investigators to refer to AD as type 3 diabetes or an “insulin resistant” condition of the brain (de la Monte, 2012; Barone et al., 2019).

Pathophysiologically, NFTs composed of aggregated and hyperphosphorylated tau are a hallmark of AD and other tauopathies (Augustinack et al., 2002; Steen et al., 2005). GSK-3β is a key serine/threonine kinase that phosphorylates tau at pathological epitopes at proline directed serine and threonine residues (Jackson et al., 2002; Johnson and Bailey, 2002). Additionally, GSK-3β inactivates glycogen synthesis and forms an important component of the insulin signaling pathway (Doble and Woodgett, 2003; Jolivalt et al., 2008). Another non-proline directed kinase that acts downstream of the insulin signaling pathway and generates pathological phospho-tau epitopes under conditions of oxidative stress is ERK (Colucci-D’Amato et al., 2003; Diehl et al., 2017). Although evidence of tau hyperphosphorylation by GSK-3β and ERK strongly indicates an impairment of the IR pathway as a risk factor in AD, the underlying mechanisms that link insulin resistance to tau hyperphosphorylation remain unclear.

The disease and severity of AD correlates strongly with the spatial and temporal progression of the insoluble aggregated tau fragments (NFTs) in the vulnerable brain regions (Didonna and Legname, 2010). Recent studies have shown that the clearance of these misfolded tau aggregates from the neurons is accomplished by autophagy (Smith, 2002; Lee, 2012). Thus, a prominent feature of AD is the massive accumulation of lysosomal vesicular structures in degenerating neurons, and pathological studies have suggested an impairment of macroautophagy in neurodegenerative disorders including AD and other tauopathies (Codogno and Meijer, 2010; Barnett and Brewer, 2011; Nassif and Hetz, 2012). Macroautophagy involves the formation of double membrane bound structures around cytosolic protein aggregates and forming autophagic vacuoles, which will subsequently fuse with the lysosomes for the degradation of protein aggregates and cellular organelles. The mTOR plays an important role in cellular homeostasis and is an inhibitor of autophagy (Jung et al., 2010). There are reports from a large body of literature, that insulin signaling leads to the activation of AKT and mTOR via a relay of phosphorylation events (Partridge et al., 2011). Phosphorylation of mTOR via the PI3K-AKT signaling pathway induces protein synthesis and downregulates autophagy, whereas dephosphorylation of mTOR has the opposite effect (Avet-Rochex et al., 2012). Several groups have shown that the PI3K/AKT/mTOR pathways are activated in the early stages of AD, while other groups have shown an upregulation of autophagy in the AD brains (Cai et al., 2012; O’Neill, 2013). However, the exact mechanism of autophagic induction and misfolded protein clearance in neurodegeneration especially with regard to insulin resistance, still remains uncertain.

*Drosophila* models misexpressing the full-length human tau have been used successfully by us and many others to recapitulate prominent features of human tauopathies that include progressive neurodegeneration and tau aggregation/phosphorylation at the disease-associated phospho-epitopes mediated by the key kinases (Jackson et al., 2002; Chatterjee et al., 2009). Likewise, human neuroblastoma cells (SHSY5Y) have been used as *in vitro* models for the study of AD and other neurodegenerative diseases (Tanaka et al., 1995; Lesort et al., 1999; Jamsa et al., 2004).

We have previously demonstrated that the misexpression of full-length (2N4R) human tau under the control of a glass promoter in the fly retina causes a marked “rough eye” phenotype with reduced eye size and missing bristles (Chatterjee et al., 2009). This “rough eye” phenotype has been instrumental in performing genetic screens to identify the modifiers of tau toxicity. Interestingly, an unbiased genetic screen conducted with the *gl*-Tau flies identified “Chico” – the single fly homolog of the mammalian IRS as one of the suppressors of the tau-induced “rough eye” phenotype (Ambegaokar and Jackson, 2011).

We therefore investigated the effect of insulin on tau phosphorylation *in vivo* by co-expressing Chico with Tau in the *Drosophila* retina. Our findings demonstrate that Chico has a strong genetic interaction with Tau, causing a suppression of tau-induced toxicity in our model. We also observe that Chico decreases the level of total tau (T46) as well as phosphorylated tau at AT8 (phospho-Ser202/Thr205) and PHF1 (phospho-Thr231/Ser235) sites. This effect correlates strongly with an elevation of inactive p-GSK-3βS9, while decreasing the level of active p-ERK (p-p44/42). We further demonstrate that these traits are reversed by Chico loss-of-function (Chico-LOF) indicating a Chico-specific effect on tau pathology. Finally, we show an increased proportion of soluble and insoluble tau aggregates in Tau transgenics that is accompanied by an induction of autophagy though not necessarily autophagic clearance. Interestingly, these effects are rescued by Chico but aggravated by Chico-LOF. We further validated our findings *in vitro* in human neuroblastoma cells under insulin resistant conditions. Collectively, these results show that the mechanisms by which insulin resistance impacts tau pathology are conserved in *Drosophila* and mammalian cell lines.

## Materials and Methods

### Stocks and Genetics

Flies were grown on Jazz mix medium (Applied Scientific Jazz Mix, Fisher Scientific, Pittsburgh, PA, United States) at 25°C. The GAL4 driver used in this study is *GMR*-GAL4 on the X chromosome. *GMR*-GAL4 (X) was placed in *trans* to *gl-*tau (A direct fusion construct of the human full-length tau cDNA to the eye specific glass promoter as described by (Jackson et al., 2002) to generate *GMR*-GAL4-*gl*tau on the X chromosome. Transgenic fly lines carrying *UAS*Chico were established by amplifying target sequences by PCR using *chico* specific primers (chico forward: 5′-ATAATTCCGCACTGGCAAAG-3′; chico reverse: 5′-CCATGCCATTAAGATGCTCA-3′) and the resulting constructs were subcloned using Exelixis (San Francisco, CA, United States) modification of *Drosophila* upstream activation sequence (UAS) expression vector (pEx-*UAS*) and microinjected into fly embryos (BestGene, Chino Hills, CA, United States). *UAS*Chico-RNAi and Chico-LOF flies were a gift from Dr. Minoru Saitoe (Tokyo Metropolitan Institute of Medical Science). *UAS*EGFP was ordered from Bloomington Fly Stock, Indiana, Stock no. 5431). The EGFP gene is located on the second chromosome.

### Light Microscopy

Light microscopy retinal images were acquired using a Nikon AZ100M light microscope (Melville, NY, United States). Extended depth of focus and volumetric images were taken using a Nikon DSFi1 camera and Nikon NIS-Elements AR 3.0 software as described previously (Chatterjee et al., 2009; Ambegaokar and Jackson, 2011).

### Histology and Immunohistochemistry

For SEM, flies were dehydrated in ethanol, incubated overnight in hexamethyldisilazane, dried under vacuum, attached to stubs with black nail polish and analyzed using a JSM-6510LV SEM (JEOL USA, Peabody, MA, United States) (Chatterjee et al., 2009). TRITC phalloidin (Sigma, St. Louis, MO, United States) whole-mount staining of adult retina was carried out as described previously (Sang and Ready, 2002). Briefly, adult retinas were dissected in cold PBS 1X pH 7.4, fixed in 4% formaldehyde/PBS for 45 min, then washed three times in PBT (0.5% Triton in PBS) and incubated for 40 min with TRITC-Phalloidin. After three washes in PBT, samples were mounted in vectashield for analysis with SP8 AOBS Laser Scanning Confocal Microscope (Leica Microsystems, Germany). 63× objective was used for scanning the retinas using a 561 nm laser spectral detector of detection bandwidth 550–650 nm.

### Immunoblotting and Reagents in *Drosophila*

Immunoblotting experiments were performed according to protocols previously described (Chatterjee et al., 2009). Briefly, about thirty freshly eclosed flies were collected and heads were decapitated and homogenized using an Argos battery-operated pestle mortar mixer for 1 min on ice in lysis buffer supplemented with complete protease inhibitors and PhosphoSTOP phosphatase inhibitor (Roche Diagnostics, Indianapolis, IN, United States). Extracts were then centrifuged at 4°C for 10 min at 11,000 × *g* and the supernatants were collected while the pellets were discarded. Samples were then mixed with an equal volume of Laemmle sample buffer with β-mercaptomethanol (Bio-Rad Laboratories, Hercules, CA, United States) and resolved by appropriate SDS-PAGE gels before transfer to nitrocellulose membranes for antibody labeling. The membranes were then incubated with appropriate secondary antibodies and developed using enchanced chemiluminiscence (ECL). Following western blot detection the membranes were re-probed with anti-β-tubulin or anti-β-actin that was used as a loading control for each experiment. The following antibodies were used, anti-tau monoclonal antibody (1:1000; T46, Invitrogen), AT8 (1:500; Thermo Scientific), anti-P-AKT505, anti-AKT, anti-mTOR, anti-P-p70S6K, and anti-P-4E-BP (1:500; Cell Signaling), anti-P-GSK-3βS9 (1:500; Genetex), GSK-3β (1:1000; United States Biologicals), anti-P-p44mapk, and p44mapK (1:500; Promega), anti-Re f(2P) (1:500; Abcam), anti-ATG8 (1:400; EMD Millipore), anti-β-tubulin, and anti-β-actin (1:2000; Sigma). The blots were quantified by ImageJ (NIH). Western blots were repeated at least three times with different sets of animals.

### Cell Culture and Treatments

SY5Y human neuroblastoma cells were ordered from ATCC and grown in DMEM supplemented with 10% heat-inactivated fetal bovine serum, 2 mM L-glutamine, 100 U/ml penicillin, and 100 μg/ml streptomycin in 5% CO_2_ atmosphere at 37°C. Cells were plated at a density of 5 × 10^3^ cells/cm^2^ in 60 mm-diameter culture dishes with 10% FBS. From day 1 after plating, cells were differentiated in the presence of 10 μm all-trans retinoic acid for a week in the cell medium containing 1% FBS. For experiments, 4 × 10^6^ cells were seeded on 60 mm dishes and cultured for 48 h (Jamsa et al., 2004). Briefly, the cells were serum-starved for 24 h and then incubated in the absence and presence of insulin (100 nm) for time periods of 10 min, 30 min, 1, 2, and 4 h, respectively.

In parallel, the cells were serum starved for 24 h and then incubated with 100 nm insulin for a period of 48 h. Post-incubation the medium was removed and supplemented progressively with 20 nm insulin for a period of 4 h, respectively. For the study of autophagic flux the cells were incubated with 100 nm bafilomycin for 4 h prior to processing. DMEM, fetal bovine serum, L-glutamine, penicillin, streptomycin, and other cell culture reagents were obtained from Life Technologies, Inc. (Invitrogen), Recombinant human insulin and *trans*–retinoic acid was from Sigma.

### Immunoblotting in SY5Y Cells and Reagents

Following insulin treatment, cells were lysed in buffer containing 50 mM Tris–HCl, pH 8.0, 150 mM NaCl, 1% Triton-X-100, 1 mM EDTA, 10 mM NaF, 1 mM Na_3_VO_4_, and one complete protease inhibitor cocktail tablet (Roche Diagnostics)/10 ml buffer. Cell lysates were centrifuged at 15,000 × *g* (Eppendorf 5417R) for 10 min. The protein content of the supernatants was measured using BCA Protein Assay kit (Pierce). An equal amount of total protein was resolved on 4–20% SDS-polyacrylamide gel electrophoresis for immunoblotting analysis using standard protocols. Primary antibodies were incubated overnight at 4°C at the following dilutions, T46 (1:1000; Invitrogen), AT8 (1:500; Thermo Scientific), pGSK-3βS9, pAKT473, mTOR, P-p70S6K, P-4E-BP1, GSK-3β, AKT, anti-IRS1-pSer616 (1:500; Cell Signaling), pERK, ERK (1:500; Promega), anti-LC3 (1:400; Novus Biologicals), and anti-GAPDH (1:500, Abcam), anti-β-actin (1:5000; Sigma). Horseradish peroxidase (HRP) conjugated secondary antibodies (Amersham Biosciences) were incubated for 1 h at room temperature in 5% milk at the dilution of 1:2000 for anti-mouse and anti-rabbit antibodies. The blots were developed by ECL.

### Immunocytochemistry and Reagents

Serum-starved SY5Y cells were stimulated at different time points with or without insulin (100 nm). After a brief PBS wash, cells were fixed for 30 min in 4% paraformaldehyde-PBS, washed three times in PBS, and blocked in 3% BSA for 1 h. Cells were then incubated with total tau antibody (A0024, DAKO) and p62 (NBL) at 1:200 dilution overnight at 4°C. After extensive washing with PBS, cells were incubated with Alexa Fluor 568 nm conjugated goat anti-mouse IgG (Invitrogen) and Alexa Fluor 488 nm conjugated goat anti-rabbit IgG (Invitrogen) and counterstained with 4, 6-diamino-2-phenylindole (1:3000 dilution) to count the number of nuclei per field of view. Cells were mounted in coverslips with vectashield (Vector laboratories). Cell analysis was carried out using a Zeiss Axioplan 2 MOT upright fluorescence microscope equipped with filter sets for DAPI, FITC, and TRITC. The microscope settings were consistent for experimental and control sets.

#### Statistical Analyses

Comparison between multiple groups in flies was done by one way ANOVA followed by Tukey-Kramer HSD test. Two experimental groups were analyzed using Student’s *t* test. Comparison between multiple groups in SY5Y cells were carried out by one way ANOVA followed by Fisher’s LSD *post hoc* test. Comparison between two experimental groups was done by Student’s *t* test. Statistical analyses were performed using Graphpad Prism or Sigma Plot software.

## Results

Several groups have shown that Chico plays a prominent role in the insulin-signaling pathway in *Drosophila* with Chico-LOF generating pronounced insulin resistance (Murillo-Maldonado et al., 2011; Naganos et al., 2012). Our previous studies from an unbiased genetic screen in *Drosophila* tauopathy models, have identified Chico as a strong modifier of the Tau phenotype. Hence, in the next sections we have investigated further the mechanistic pathways by which this interaction occurs.

### Misexpression of Chico in the Fly Eye Ameliorates Human Tau Induced Rough-Eye Phenotype

**FIGURE 1:**
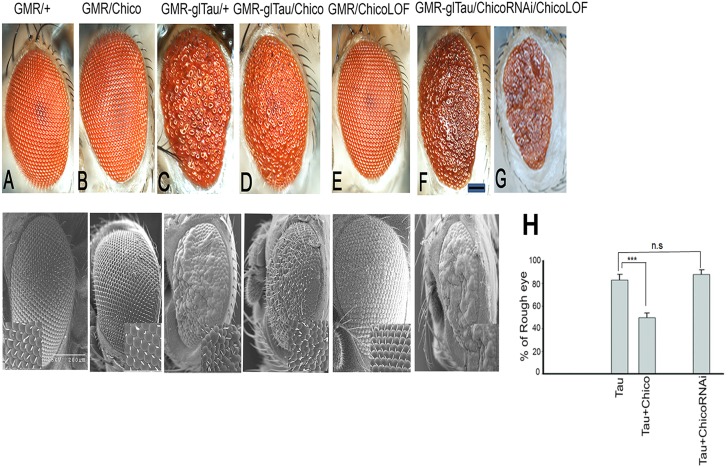
Overexpression of Tau induces a rough eye phenotype that is partially suppressed by Chico coexpression; conversely Tau coexpressed with Chico-RNAi or Chico-LOF restores the rough eye phenotype. **(A–G)** Displayed are photomicrographs and scanning electron microscopy of progeny eyes taken 24–48 h post-eclosion; **(A)** driver alone, **(B)** Chico, **(C)** Tau, **(D)** Tau+Chico, **(E)** Chico-RNAi, **(F)** Tau+Chico-RNAi, **(G)** Tau+Chico-LOF (null allele). Scale bars, 50 μm for the light micrographs and 200 μm for the scanning electron micrographs. **(H)** Quantitation of the percentage of “Rough eye” phenotypes in Tau, Tau+Chico, and Tau+ChicoRNAi transgenics. Error bars represent SEM. *N* = 15 eyes/genotype, ^∗∗∗^*P* < 0.001 relative to Tau flies. Student’s *t* test was done for pairwise comparison between two groups.

To investigate how insulin signaling impacts tau pathology, we utilized our well-established model of Tauopathy in which we expressed full-length wild type human Tau (2N4R) in *Drosophila* eyes using the pan-retinal *GMR*-GAL4 driver (Jackson et al., 2002; Chatterjee et al., 2009). As reported previously, Tau misexpression in the eye resulted in smaller eye size with a rough anterior and missing bristles (Figure 1C). Interestingly, co-expression of Chico with Tau ameliorated the “roughness” of the eye phenotype, resulting in larger eyes with fewer missing bristles (Figure 1D) while ChicoRNAi or Chico-LOF (null allele chico[1]) with Tau, resulted in a more severe worsening of the “rough-eye” phenotype (Figure 1F,G). As compared to the double transgenics, the Control eyes of (*GMR*/+), *GMR*/*UAS*Chico and *GMR*/*UAS*ChicoRNAi appeared perfectly normal (Figure 1A,B,E). Magnification of the SEM insets (400X) clearly demonstrates fused ommatidia and missing bristles in Tau flies (Figure 1C, lower panel) that are partially rescued by Chico (Figure 1D, lower panel) and exacerbated by Chico-LOF (Figure 1F, lower panel). Quantification of the percentage of rough area per eye in each genotype revealed an 80% rough-eye area in Tau and Tau+Chico-LOF dual transgenics as compared to 45% rough-eye area in Tau+Chico transgenics compared to Controls (Figure 1H, lower panel). In contrast, *UAS*EGFP co-expressed with Tau, failed to suppress the “rough-eye” phenotype confirming that the effect of Chico on Tau is specific (Supplementary Figure S1A).

In order to compare the internal retinal morphology, confocal imaging of adult retinas stained with TRITC-phalloidin was performed. Tangential optical sections display a normal trapezoidal array of rhabdomeres in the driver-alone controls (Supplementary Figure S2A). In contrast, Tau flies produced disorganized ommatidia with massive loss of photoreceptor neurons (Supplementary Figure S2B). The retinas of transgenics co-expressing Chico (Supplementary Figure S2C) showed a relatively normal array of rhabdomeres other than some abnormal polarity.

Taken together our results show that the co-expression of Chico with Tau atleast partially rescues tau-induced neuronal loss and protects against tau-induced neurotoxicity.

### Misexpression of Chico With Tau Reduces the Levels of Total and Hyperphosphorylated Tau

We and others have previously shown that the “roughness” of the eye phenotype in Tau transgenics is associated with elevated tau expression as well as hyperphosphorylation at the disease epitopes (Jackson et al., 2002; Chatterjee et al., 2009). Therefore, in order to analyze the rescue of the “rough-eye” phenotype in Tau+Chico flies, we examined if there were any alterations in the levels of total and hyperphosphorylated tau in these transgenics.

**FIGURE 2:**
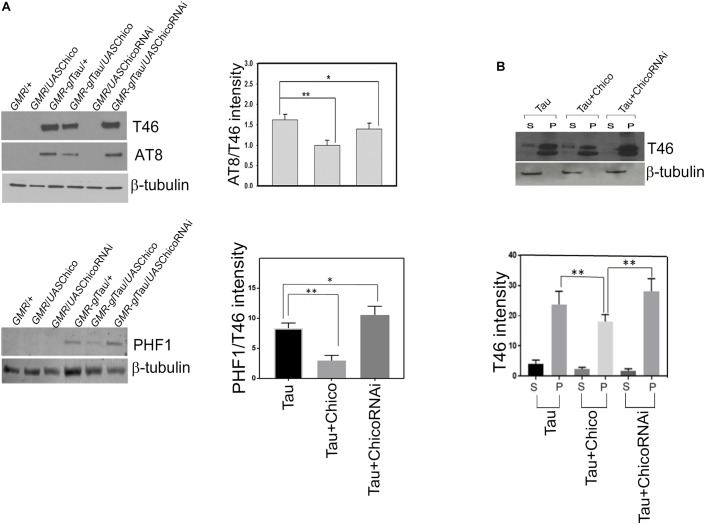
Western blot data shows a decrease in the levels of total (T46) and phosphorylated tau (AT8, PHF1) when Chico is coexpressed with Tau; the T46 and AT8 levels are reversed when Chico-RNAi is coexpressed with Tau. **(A)** Representative blots are probed with T46 (total tau), AT8, PHF1, (phospho-tau), and β-tubulin antibodies, respectively. All flies were 3 days after eclosion. Genotypes are as follows: *GMR*-GAL4/+; *GMR*-GAL4/*UAS*Chico; *GMR*-GAL4-*gl-*Tau/+; *GMR*-GAL4-*gl-*Tau/*UAS*Chico; *GMR*-GAL4/*UAS*Chico-RNAi; *GMR*-GAL4-*gl-*Tau/*UAS*Chico-RNAi. **(B)** Tau and Chico coexpression decreases the level of sarcosyl soluble and insoluble tau aggregates in the supernatant and pellet as compared to Tau alone and Tau+Chico-LOF dual transgenics. Western blot analysis from flies probed with T46 antibody against total tau. Genotypes: *GMR*-GAL4-*gl*-Tau/+; *GMR*-GAL4-*gl*-Tau/*UAS*Chico; *GMR*-GAL4-*gl*-Tau/*UAS*Chico-LOF. Densitometric analyses of AT8/T46 and PHF1/T46 levels and total tau distribution in soluble and pellet fractions are normalized to β-tubulin. (One way ANOVA with Tukey-Kramer HSD was used for multiple groups comparison or *post hoc* Dunnett’s test for comparison against Tau, *n* = 3 sets of biological replicates for each genotype.) Data represents the mean ± SEM, ^∗∗^*P* < 0.01 and ^∗^*P* < 0.05 relative to Tau.

Our results show that coexpression of Chico and Tau significantly decreased the total tau (T46) and phospho-tau at AT8 and PHF1 residues leading to >50% reduction of AT8/T46 and PHF1/T46 ratios as compared to Tau-alone flies. We further observed that these effects were reversed when Tau was coexpressed with Chico-LOF or ChicoRNAi (Figure 2A and Supplementary Figure S3A). Our data also showed that the coexpression of EGFP produced no alterations in the tau levels confirming that this is a Chico-specific effect (Supplementary Figure S1B). In summary, our results suggests that the ability of Chico to ameliorate the tau-induced “rough-eye” phenotype occurs by decreasing the level of total and phospho-tau at pathological epitopes.

### Misexpression of Chico With Tau Decreases the Formation of Soluble and Insoluble Tau Aggregates Compared to Tau and Tau+ChicoRNAi

Aggregation of hyperphosphorylated tau is one of the key features of AD and related tauopathies (Simic et al., 2016). Our previous studies in tauopathy models demonstrated the accumulation of sarcosyl soluble and –insoluble tau aggregates that contributed toward tau-induced neurotoxicity (Chatterjee et al., 2009). In line with these reports, we investigated whether loss or GOF of Chico has any effect on sarcosyl solubility of tau. Co-expression of Chico with Tau reduced the accumulation of sarcosyl-soluble and insoluble tau species in the supernatant and pellet fractions, respectively. However, co-expression of ChicoRNAi significantly altered the pattern of tau solubility and increased accumulation of sarcosyl-insoluble tau (Figure 2B). Although recent studies have demonstrated that soluble tau aggregates are toxic for neuronal cells, sarcosyl-insoluble tau aggregates eventually form the NFTs detected in AD brains (Ren and Sahara, 2013). Taken together, our data shows that in addition to decreasing tau expression and hyperphosphorylation, Chico reduces the formation of sarcosyl-soluble and insoluble tau aggregates in Tau transgenics.

### Effect of Chico on Tau Hyperphosphorylation Is Mediated by GSK-3β

Given that the insulin signaling pathway in *Drosophila* is highly conserved with that of higher vertebrates, and the kinases GSK-3β and ERK lie downstream of Chico in the insulin signaling cascade; we investigated whether there is a direct effect of Chico on the regulation of these kinases in our model.

**FIGURE 3:**
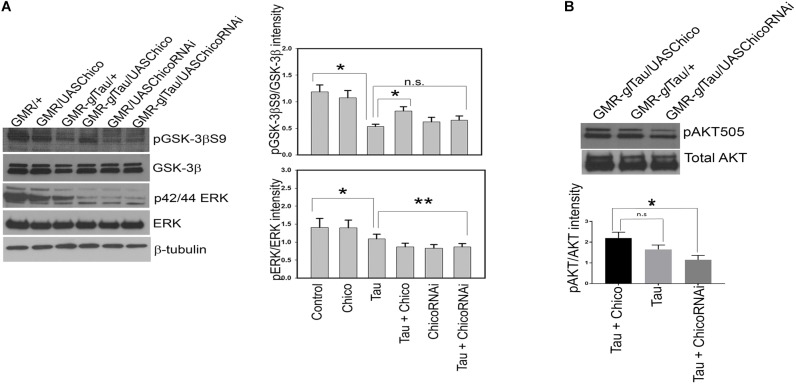
Overexpression of Tau decreases pGSK-3βS9 and pERK in representative blots. Coexpression of Chico restores the levels of pGSK-3βS9 but not of pERK. **(A)** Western blot analysis from flies probed with pGSK-3βS9, pERK, total GSK-3β, total ERK, and β-tubulin antibodies, respectively. **(B)** Western blot analysis from flies probed with pAKT and total AKT antibodies, respectively. Quantification of phospho-antibody levels are normalized to total protein levels. (One way ANOVA with Tukey-Kramer HSD was done for multiple group comparisons or *post hoc* Dunnett’s test was used for comparison with Tau, *n* = 3 sets of biological replicates for each genotype.) Data represents the mean ± SEM, ^∗∗^*P* < 0.01 and ^∗^*P* < 0.05 relative to Tau in panel **(A)** and to Tau+Chico in panel **(B)**. All flies were 3 days before eclosion. Genotypes: *GMR*-GAL4/+; *GMR*-GAL4/*UAS*Chico; *GMR*-GAL4-*gl*-Tau/+; *GMR*-GAL4-*gl*-Tau/*UAS*Chico; *GMR*-GAL4/*UAS*Chico-RNAi; *GMR*-GAL4-*gl*-Tau/*UAS*Chico-RNAi in panel **(A)**. Genotypes: *GMR*-GAL4-*gl*-Tau/*UAS*Chico; *GMR*-*gl*-Tau; and *GMR*-*gl*-Tau/*UAS*Chico-RNAi in panel **(B)**.

Our data showed a >50% reduction in the level of inactive p-GSK-3βS9 in Tau genotypes compared to controls (GMR/+ and GMR/*UAS*Chico) that matched with the elevated tau phosphorylation at AT8 and PHF1 epitopes. Co-expression of Chico with Tau rescued the levels of phospho-GSK-3βS9 while Tau+ChicoRNAi lines reversed the effect (Figure 3A). Conversely, when active GSK-3β (phospho-GSK-3βY216) levels were assessed; Tau+Chico transgenics showed a marked reduction in active GSK-3β compared to Tau-only and Tau+ChicoRNAi transgenics (data not shown).

Next, we analyzed the phosphorylation status of active ERK (p42/44) and observed <10% reduction in Tau transgenics compared to Controls. Interestingly, this effect was not rescued by either Chico overexpressed or knock-down lines (Figure 3A). Despite the alterations in phosphorylated GSK-3β and ERK, total protein levels remained constant in all the genotypes.

Collectively, our data suggests that Chico-mediated effects of tau hyperphosphorylation display a direct correlation with GSK-3β but not with ERK.

To assess whether insulin resistance was generated by the gain or LOF of Chico, we analyzed the ability of Chico to stimulate AKT phosphorylation at position Serine505. This phospho-AKT site in flies is analogous to mammalian AKT phosphorylated at Serine473 and a marker of insulin sensitivity (Musselman et al., 2011). We observe that compared with other genotypes, the Tau+ChicoRNAi lines show a 50% reduction in phospho-AKTSerine505 levels suggesting an insulin-resistant phenotype (Figure 3B).

### Tau-Induced Down-Regulation of TOR Pathway Components Is Rescued in Tau+Chico Double Transgenics

**FIGURE 4:**
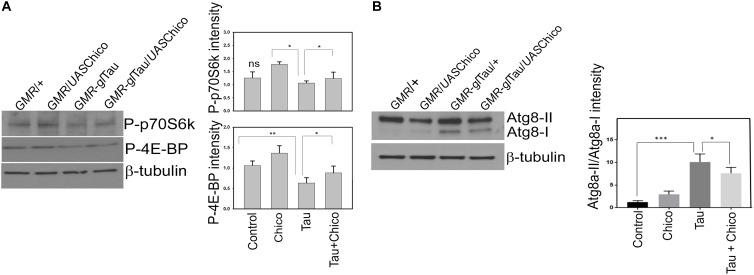
Tau overexpression effects a reduction in the level TOR pathway markers but an increase in autophagy pathway markers (Atg8a-II/Atg8a-I) that are rescued by Chico coexpression with Tau. **(A,B)** Western blot analysis from flies probed with P-p70S6K, P-4E-BP1, and Atg8 antibodies. β-tubulin is the loading control. Quantification of the levels of the antibodies normalized to β-tubulin levels. (One way ANOVA with Tukey-Kramer HSD was done for multiple group comparisons or *post hoc* Dunnett’s test was done for comparison with Tau, *n* = 3 sets of biological replicates for each genotype.) Data represents the mean ± SEM, ^∗∗∗^*P* < 0.001, ^∗∗^*P* < 0.01, and ^∗^*P* < 0.05 relative to Tau. All flies were 3 days after eclosion. Genotypes: *GMR*-GAL4/+; *GMR*-GAL4/*UAS*Chico; *GMR*-GAL4-*gl-*Tau/+; and *GMR*-GAL4-*gl-*Tau/*UAS*Chico.

As in higher vertebrates, in *Drosophila*, the insulin signaling pathway is upstream of IRS/PI3K pathway, which in turn positively regulates target of rapamycin (TOR) activity. In a healthy environment, TOR activates protein synthesis and promotes cellular growth and proliferation by enhancement of important translational components, including the translation initiation factor 4E binding proteins (4E-BP1-3) and ribosomal protein S6 kinases. However, under stressful conditions, TOR signaling is impaired and autophagy is upregulated (Cai et al., 2012; O’Neill, 2013). Interestingly in our studies, we observed a 40% reduction of the TOR pathway components phospho-p70S6K and phospho-4E-BP1 in the Tau flies with a partial rescue observed in Tau+Chico transgenics (Figure 4A). In addition, we assessed the TOR protein levels in the Tau flies. A significant reduction of phospho-TOR/Total TOR was found in Tau flies compared to Control and Chico-only flies. This effect was rescued partially by Chico co-expression (Supplementary Figure S4A).

### Upregulation of Autophagy Is Observed in Tau and Tau+ChicoRNAi Transgenics

Previous studies have demonstrated that the inactivation of the TOR pathway stimulates basal autophagy (Hands et al., 2009; Metcalf et al., 2012; O’Neill, 2013). The *Drosophila* homologs of mammalian autophagy marker LC3 are Atg8a and Atg8b. Atg8a particular, has been extensively used as an autophagy marker in *Drosophila*. The lipidation of Atg8a produces Atg8a-II that is directed to the autophagosomes as opposed to Atg8a-I, which is the unprocessed Atg8a protein (Nagy et al., 2015). Compared to other genotypes Tau transgenics showed significant upregulation of autophagy that was partially reduced by Chico. Interestingly, Tau+Chico-LOF flies displayed a similar enhancement of autophagy (Figure 4B and Supplementary Figure S5).

However, an increase in Atg8a-II signal implies an enhanced autophagic induction but not necessarily an autophagic clearance (Rusten and Stenmark, 2010). Hence we decided to investigate if there was an alteration in the levels of Ref(2P) – the fly homolog of mammalian p62 which accumulates due to an impairment in autophagy (Son et al., 2012). Although there was a moderate increase of Ref(2P) accumulation in Tau and Tau+Chico-LOF, it was not significant compared to other genotypes (data not shown). Thus, in summary, our data suggests that Tau overexpression induces autophagy without an autophagic blockage.

### Progressive Insulin Treatment of SY5Y Cells Causes a Time-Dependent Increase in Total and Phosphorylated Tau That Correlates With the Decrease of Inactive GSK-3β

**FIGURE 5:**
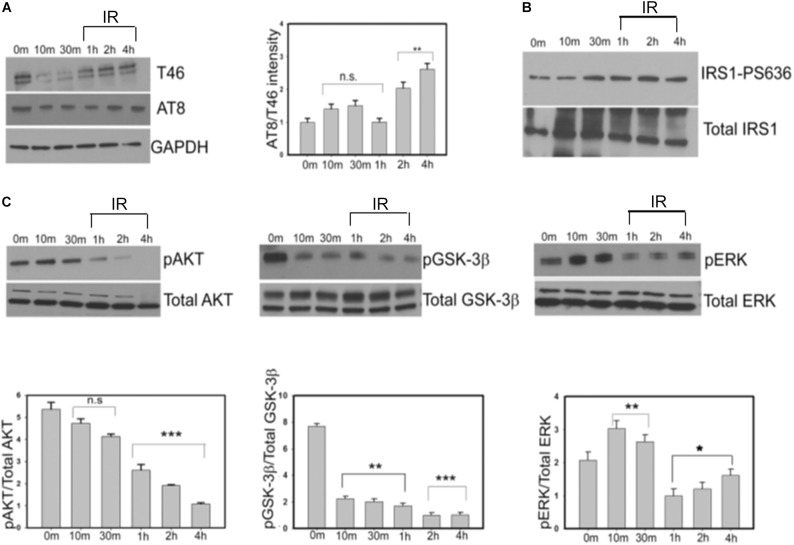
Progressive insulin treatment increases total and phosphorylated tau in SY5Y cells in an insulin resistant state. This is accompanied by the gradual decrease in the levels of pAKT and pGSK-3βS9. SY5Y cells were serum-starved for 24 h and then treated with 100 nm insulin for various time points (0, 10, 30, 60, 120, and 240 min). After treatment, cells were harvested, lysed and immunoblotted with either **(A)** anti-tau (T46) and AT8 antibodies **(B)** IRS1-pS636 and total IRS1 or **(C)** pAKT, pGSK-3β, pERK, and total kinase antibodies in the representative blots. GAPDH was used as a loading control. Densitometric analyses of phosphorylated tau (AT8) over total tau (T46) and phosphorylated kinases (pAKT, pGSK-3β, and pERK) over total kinases normalized to GAPDH were performed based on four independent experiments. Data represent the mean ± SEM, ^∗∗∗^*P* < 0.001, ^∗∗^*P* < 0.01, and ^∗^*P* < 0.05 versus control (0 min), *n* = 4 biological replicates. (One way ANOVA followed by Fisher’s test for multiple comparisons and Student’s *t* test for pairwise comparisons.) IR, insulin resistance.

To determine whether the insulin-induced alteration in the total and phospho-tau levels that we observe in our *Drosophila* tauopathy models, is conserved in human cells, we next investigated the effect of insulin treatment in neuroblastoma cells. SY5Y cells were serum-starved for a period of 24 h and then treated with 100 nm insulin for 8 h (Son et al., 2012). We observed an immediate decrease in T46 (total Tau) and AT8 (Serine 202/Threonine 205) that lasted for a period of 30 min of insulin treatment, followed by a gradual increase in the levels of total and AT8-tau over a period of 4 h after which it remained constant. However, as the insulin treatment continued for a period of 4 h, AT8-tau levels were significantly elevated as compared to total tau (T46), thereby increasing the ratio of AT8/T46 by almost 2.5-fold (>50%) at 4 h as compared to controls (0 min post-treatment) (Figure 5A and Supplementary Figure S3B).

Next, we examined the change in phospho-IRS-1(Ser636) at specified time intervals to monitor insulin resistance in the cells. From 30 min to 4 h we observed a gradual increase in the ratio of phospho-IRS1(Ser636)/Total IRS1 levels consistent with an insulin-resistant phase in our cellular model (Figure 5B).

Insulin resistance in animal and cellular models of type 2 diabetes is also marked by a reduction in phospho-AKTSer473 while total AKT remains constant (Shao et al., 2000; Standaert et al., 2002). Our data showed that phospho-AKTS473 levels did not change significantly compared to control till a period of 30 min. However, post-insulin treatment there was a gradual decrease of phospho-AKT/Total AKT signal from 1 h (50% reduction) to 4 h (80% reduction) signifying progressive insulin resistance (Figure 5C, left panel). Taken together our results indicate a moderate increase in the level of total tau and a more substantial increase in phospho-tau in a progressively insulin-resistant environment.

To determine whether alterations in tau phospho-epitopes correlate with changes in the key kinases downstream of the IR pathway, we next examined the levels of GSK-3β and ERK using phospho-specific antibodies over a period of 4 h. We observed a progressive decrease in phospho-GSK-3βS9/Total GSK-3β levels to 80% compared to controls at the end of 4 h. This correlated with the gradual increase in the AT8 immunoreactivity that we observed earlier. On the contrary, the phospho-ERK/Total ERK immunoreactivity did not change significantly at the end of 4 h. In addition, we did not detect any change in the level of total GSK-3β or ERK, indicating that the change in the pattern of phosphorylated kinases is an insulin signaling pathway-specific response (Figure 5C, middle and right panels).

In summary, our data indicates that tau hyperphosphorylation maybe contributed by active GSK-3β but not by active ERK in insulin-treated cells.

### Insulin Treatment of SY5Y Cells Progressively Affects the Levels of Protein Synthesis Regulators in the mTOR Pathway

**FIGURE 6:**
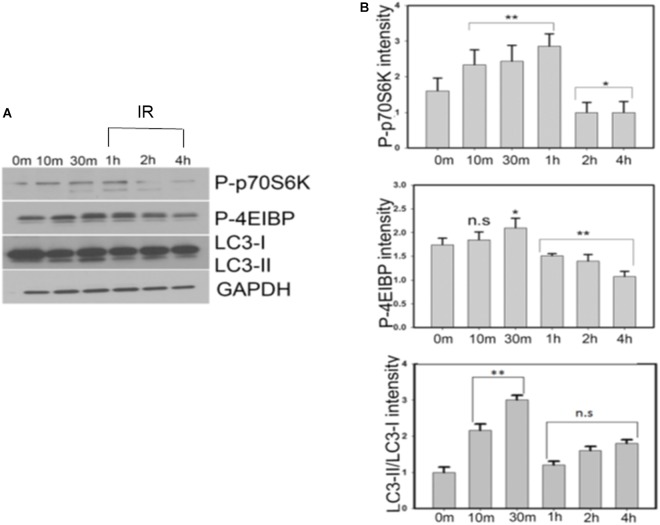
Insulin treatment of SY5Y cells affects the levels of autophagic markers via mTOR pathway demonstrated by a time dependent decrease in the levels of p-p70 S6K and p-4E-BP1 proteins. A simultaneous increase in the level of LC3-II/LC3-I ratio is observed till 30 min after which there is a decrease of autophagy in the insulin-resistant phase. **(A)** SY5Y cells were treated with 100 nm insulin for different time points (0, 10, 30, 60, 120, 240 min) and lysates were analyzed by immunoblotting with antibodies against P-p70S6K, P-4E-BP1, and LC3. GAPDH was used as a loading control. **(B)** Densitometric analysis of these antibodies relative to GAPDH was performed based on four independent experiments. Data represent the mean ± SEM, ^∗∗^*P* < 0.01, ^∗^*P* < 0.05 versus control (0 min), *n* = 4 biological replicates (One way ANOVA followed by Fisher’s test for multiple comparisons and Student’s *t* test for pairwise comparisons.) IR, insulin resistance.

It has been previously reported that in the normal physiological environment and in the presence of sufficient nutrients, insulin or insulin-like growth factors activate PI3K/AKT/mTOR pathways subsequently promoting protein synthesis and inhibiting autophagy (Hands et al., 2009; O’Neill, 2013). We observed that insulin treatment of SY5Y cells initially increased the levels of phospho-mTOR (Ser2448), phospho-p70S6K and phospho-4E-BP1 for a period of 1 h followed by a progressive decrease in the levels of protein biosynthesis markers as the cells gradually entered an insulin-resistant phase at the end of the 4-h time-period. Conversely, the ratio of autophagic marker LC3II/LC3I increased rapidly within the first 30 min of insulin treatment, indicating an activation of autophagy. However, at the end of 4 h of insulin treatment, the LC3II/LC3I ratio significantly reduced compared to earlier time points (10 and 30 min), suggesting a blockage in the autophagic pathway (Figure 6 and Supplementary Figure S4B). These results indicate that progressive insulin stimulation not only alters the levels of tau and key kinases but also mediates changes in protein synthesis and key autophagic markers in our model.

### Insulin Pre-treatment of SY5Y Cells Increases the Tau Levels by Autophagic Impairment

**FIGURE 7:**
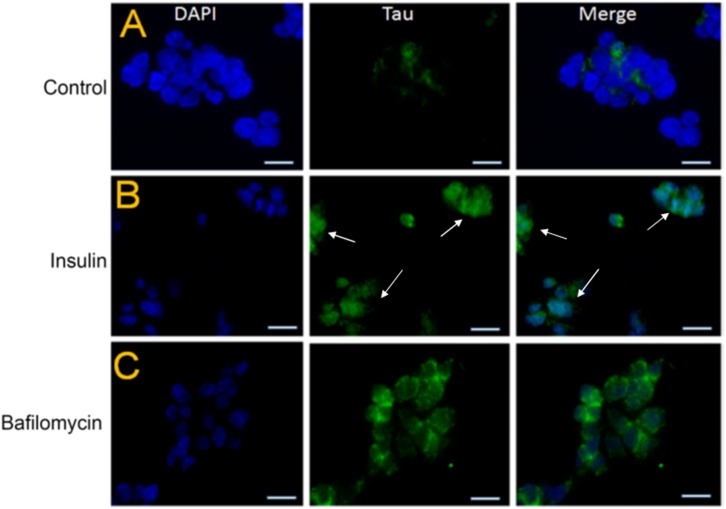
Pre-treatment with insulin increases the tau protein levels in SY5Y cells relative to untreated controls. **(A–C)** SY5Y cells were serum-starved for 24 h and then treated with 100 nm insulin for 48 h followed by progressive treatment with 20 nm insulin at various time points (0, 1, 5, 10, 30, 60, 120, and 240 min) In parallel, SHSY5Y cells were subjected to autophagy-blocker Bafilomycin treatment for 4 h. This was followed by immunostaining by total Tau antibody (green) and DAPI (blue). Tau immunoreactivity increased in the insulin and bafilomycin-treated cells compared to untreated controls (bar = 20 μm). White arrowheads represent total Tau in insulin-treated cells.

To further investigate whether tau accumulation in insulin-treated SY5Y cells was linked to an impairment of autophagy, we pre-treated these cells with 100 nm insulin for a period of 48 h after serum starvation and exposed them to 20 nm insulin progressively for a period of 4 h (Son et al., 2012). In parallel, these SY5Y cells were also exposed to bafilomycin treatment. Bafilomycin is one of the well-known compounds that inhibit late-stage autophagy and prevents maturation of autophagic vacuoles by impairing the fusion between autophagosomes and lysosomes (Mauvezin and Neufeld, 2015). Interestingly, we observed that pre-treatment of the SY5Y cells with insulin and bafilomycin enhanced tau staining (A0024) compared to untreated controls (Figure 7B,C vs. Figure 7A).

**FIGURE 8:**
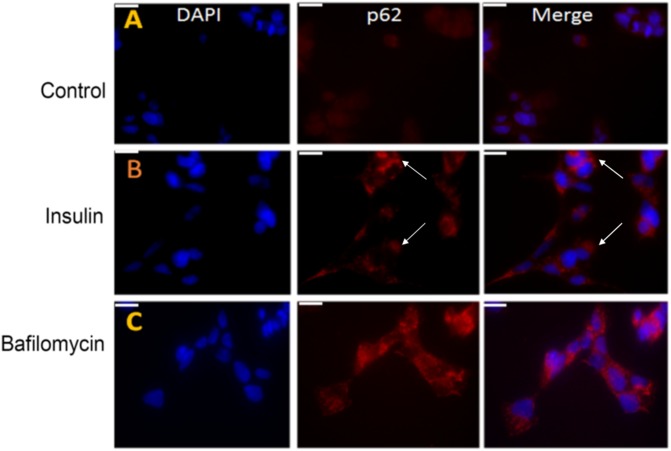
Pre-treatment with insulin increases the p62 protein levels in SY5Y cells relative to untreated controls. **(A–C)** SY5Y cells were serum-starved for 24 h and then pre-treated with 100 nm insulin for 48 h, followed by immunostaining with p62 (red) antibody. In parallel, SHSY5Y cells were subjected to autophagy-blocker bafilomycin treatment for 4 h. The intensity of p62 was greater in insulin and bafilomycin-treated cells **(B,C)** compared to untreated cells **(A)** as observed by fluorescence microscopy implying an autophagic blockage. DAPI (blue) was used as a nuclear stain (bar = 20 μm). White arrowheads represent p62 in insulin-treated cells.

Previous studies have also shown that the lack of autophagy or blockage of autophagic flux leads to the accumulation of p62 protein in cells (Rusten and Stenmark, 2010). Immunohistochemical studies in SY5Y cells also showed an increased accumulation of p62 (red) in the bafilomycin-treated cells implying an impairment of autophagy. A similar p62 enhancement was observed in the insulin-treated cells confirming an inhibition of autophagy or a blockage of autophagic flux (Figure 8B,C vs. Figure 8A).

Taken together our results show that pre-treatment of SY5Y cells with insulin results in an inhibition of autophagy and increased tau protein accumulation. It is worthwhile to note that SY5Y cells treated with both insulin and bafilomycin were toxic resulting in massive cell death possibly due to a combination of tau aggregation and autophagic impairment triggering apoptotic pathways.

## Discussion

Alzheimer’s disease is the most common cause of dementia in humans (Citron, 2004). While plaques and tangles are designated as pathological signatures of AD, it is now speculated that these factors are consequential rather than causal of the neurodegenerative cascade (Ishiguro et al., 1992; Armstrong, 2006). Despite intense investigation, the etiology and pathogenesis of sporadic AD remain unknown. Brain glucose metabolism is impaired in AD, and growing evidence supports the concept that AD is fundamentally a metabolic disease with progressive dysfunction in brain glucose utilization and responsiveness to insulin and insulin-like growth factor stimulation (Freude et al., 2009; Schuh et al., 2011). Although, there have been numerous studies on peripheral insulin resistance, the mechanism of central insulin resistance and particularly its impact on tau, one of the hallmarks of AD, is relatively unknown.

Our laboratory has generated a model of tauopathy in *Drosophila* by expressing human wild-type full-length tau (2N/4R) in the retina, that results in a “rough-eye” phenotype (Jackson et al., 2002; Chatterjee et al., 2009). This tau-induced eye phenotype is highly useful for conducting unbiased enhancer/suppressor screens through which Chico-the single fly homolog of mammalian IRS was identified as a suppressor of tau-induced “rough-eye” phenotype (Ambegaokar and Jackson, 2011). Recent studies show that loss-of-function mutation in Chico causes insulin resistance by increasing the amount of circulating trehalose and lipids in *Drosophila* (Murillo-Maldonado et al., 2011; Naganos et al., 2012). Our data demonstrates that Chico ameliorates the tau-induced neurotoxicity by reducing tau aggregation and hyperphosphorylation at the disease epitopes while Chico-LOF exacerbates these effects. The exacerbation of tau pathology by Chico-LOF was attributed to insulin resistance as these flies displayed 50% reduction in *Drosophila* AKT phosphorylated at Serine505 residue that corresponds with insulin resistance in mammalian AKT phosphorylated at Serine473 residue (Standaert et al., 2002). Our results further show that this augmentation is achieved by modulating the kinase GSK-3β, which lies downstream of the IR signaling pathway. Finally, we demonstrate that Chico impacts mTOR/autophagy pathway, thus playing a significant role in clearance of tau aggregates. These observations in the *Drosophila* tauopathy model were further validated in mammalian neuroblastoma cells, implying a conservation of these pathways across diverse systems.

In post-mortem brains from AD patients, tau phosphorylation was found to increase at several GSK-3β directed epitopes such as AT8, AT180, AT100, and PHF1 (Ishiguro et al., 1992; Armstrong, 2006). GSK-3β is one of the key tau kinases that also colocalizes with tau tangles and microtubules in the brains from patients with AD (Hooper et al., 2008; Avila et al., 2012). Interestingly, GSK-3β is regulated by the insulin signaling pathway. Briefly, insulin signaling is initiated by the binding of insulin to its receptor, located in the cytoplasmic membrane. This leads to rapid phosphorylation of IRSs that activate PI3K/AKT signaling in the brain (Diehl et al., 2017). AKT phosphorylates GSK-3β at Serine 9, thereby inhibiting its activity of tau hyperphosphorylation and facilitating binding to microtubules. Conversely, when GSK-3β is in an active state (due to phosphorylation at Tyrosine 216 residue), it phosphorylates tau to generate AT8 and PHF1 disease epitopes (Chatterjee et al., 2009). In another independent study Barone et al. have shown that reduction of Biliverdin reductase-A (BVR-A) in the hippocampus of 3xTg-AD mice impairs AKT-mediated inhibition of GSK-3β and increases tau phosphorylation in response to oxidative stress. They have also shown that increased GSK-3β activation (decreased Serine 9 phosphorylation) is detected in the early stages of AD (Sharma et al., 2019).

In our study we observe, that co-expression of Chico with Tau significantly increases the level of inactive GSK-3β phosphorylated at Serine9 residue and reduces tau hyperphosphorylation. Conversely, the coexpression of insulin-resistant Chico-LOF activates GSK-3β and exacerbates tau hyperphosphorylation. Our results are supported by the studies of Jolivalt et al. (2008) and Kim et al. (2009) in which STZ-induced type-1 diabetic mouse models display an increased tau hyperphosphorylation mediated by active GSK-3β (Doble and Woodgett, 2003; Kim et al., 2009). Interestingly this effect was not observed in type 2 diabetic mouse models. Our study differs significantly in this respect since the genetically manipulated Chico-LOF mutants recapitulate features of type 2 diabetes and not of type 1 diabetes.

To examine whether insulin-resistance – induced tau enhancement was conserved in the mammalian system, these experiments were repeated in insulin treated SY5Y cells. It has been observed in human hippocampal tissue that insulin resistance is accompanied by an increased phosphorylation at the Serine636 residues of IRS-1 (Copps and White, 2012; Diehl et al., 2017). Interestingly we observed a progressive increase in the ratio of phospho-IRS1/Total IRS1 from 30 min to 4 h time period signifying insulin resistance. This was also confirmed by an 80% reduction of phospho-AKT/Total AKT at the 4 h time-period confirming insulin resistance in SY5Y cells.

Interestingly, while we observed an initial decrease in total and AT8-tau levels, there was a sharp increase in the AT8/T46 ratios as the cells entered an insulin resistant condition. Our studies are in contrast to the studies done by Lesort et al. (1999) who treated the SY5Y cells with 10 nm insulin and observed a transient increase in AT8-tau hyperphosphorylation at the early time points (0–60 min) and dephosphorylation post 1 h. In comparison, we have used 100 nm insulin which generated insulin resistance after 30 min of treatment subsequently increasing levels of total and phospho-tau. In this respect, the early time points in SY5Y cells recapitulate our observations from Chico gain-of-function while the later time points pertaining to insulin-resistance represent Chico loss-of-function or knock-down flies.

Several studies have shown that hyperphosphorylated tau dislodges from the microtubules and binds with tau monomers to form tau aggregates. These abnormal tau aggregates are considered to be a critical pathological feature of tauopathy (Cowan et al., 2015; Goedert and Spillantini, 2017). It is hypothesized that small molecular weight, sarcosyl-soluble tau aggregates gradually consolidate to form NFTs that are sarcosyl-insoluble. However, the toxicity of the sarcosyl soluble and insoluble aggregates remains controversial. A study by Lasagna-Reeves et al. (2011) showed that the injection of soluble tau oligomers but not monomers or fibrils were sufficient in inducing synaptic dysfunctions and cognitive impairments in wild type mouse. There are others studies that show the spread of soluble tau across the synapses for the propagation of tau pathology while the insoluble tau fibrils within the neurons act as a “sink” to sequester the toxic and soluble tau species (Kopeikina et al., 2012). Although, Chico-LOF exacerbates the tau-induced toxicity in the “rough-eye” phenotype, we detect a significant amount of insoluble tau species in these transgenics. Taking into account the prevailing literature in this area, there is a possibility that the shift of tau from soluble to insoluble aggregates in an insulin-resistant state is a defense mechanism of neurons in response to progressive tau accumulation.

Aggregates of tau are removed by macroautophagy (Inoue et al., 2012; Neufeld, 2012). The protein kinase TOR, an evolutionarily conserved protein, plays a central role in regulating macroautophagy in response to nutrient availability and stress factors (Miron et al., 2003). The involvement of TOR pathway in *Drosophila* neurodegeneration models have been controversial depending on disease model in question. Feany and colleagues and Berger et al. (2006) have previously reported that TOR pathways components enhance mutant tau (R406W) induced toxicity in their fly models that is ameliorated by treatment with TOR inhibitor rapamycin (Khurana et al., 2006; Khurana et al., 2010). These observations have also been supported by Caccamo et al. (2013) in P301S mouse models where increasing mTOR activity has been shown to increase tau pathology whereas decreasing mTOR ameliorates tau-induced neuronal dysfunctions and pathology. In contrast, we observe a down-regulation of the TOR pathway in our tauopathy models that is rescued by coexpression of Chico. One possibility is that we have used wild type full-length human tau in our fly models as opposed to mutant tau used in the studies above. An interesting study by Tramutola et al. (2015) in post-mortem AD brains reports significant upregulation of PI3K/AKT/mTOR pathways in both MCI and late stage AD but not in the early stage AD. Since we have used 0–3 days old adult flies for this study, it is possible that our tauopathy model represents an early stage of pathology during which the PI3K/AKT/mTOR signaling pathway is not yet activated.

In keeping with decreased TOR function, we observe a simultaneous upregulation of autophagy in Tau transgenics. This was measured by elevated levels of membrane bound Atg8a-II relative to unbound Atg8a-I. We also observe a similar upregulation of autophagy in Tau+Chico-LOF transgenics. However, an upregulation of autophagy does not necessarily mean autophagic clearance. A recent study by Bakhoum et al. (2014) demonstrates the formation of giant autophagic bodies (GABS) in retinas of tauopathy models that arise due to incomplete acidification of autophagolysosomes and culminates in an impairment of autophagic flux. Although we did not see an autophagic blockage in our freshly eclosed progeny, further experiments need to be done with aged Tau flies. The relationship between insulin resistance and autophagy is a relatively new field with contradicting observations depending on animal or cellular model in question. Recent studies in cellular models of carcinoma show that insulin resistance activates autophagy (Zhou et al., 2009). In contrast, studies done in APP/PS1 mice show that insulin sensitivity improves autophagy in neurodegenerative disease models (Macklin et al., 2017). Thus, in our Tauopathy model, the impact of hyperphosphorylated and aggregated tau on autophagic clearance in an insulin-resistant environment cannot be ruled out (Rodriguez-Rodriguez et al., 2017).

In SY5Y cells, prolonged treatment with insulin resulted in downregulation of the mTOR pathway in an insulin-resistant environment. Interestingly, our data matches with the observations of Barone et al. (2016) who report mTOR activation at lower concentrations of insulin but a subsequent decrease in higher insulin concentrations in SY5Y cells. However, instead of an upregulation of autophagy, we observed an autophagic blockage that was evident by decreased LC3-II signaling in insulin-treated cells compared to controls. These results were further validated by the accumulation of p62 protein in insulin-treated cells indicating an autophagic blockage akin to the treatment of the cells with known autophagy blocker Bafilomycin (Bartlett et al., 2011; Cai et al., 2012). It is possible that the autophagic upregulation observed at the early time points was atleast partly contributed by the serum starvation before subjecting the cells to insulin treatment.

Taken together our results imply that insulin resistance increases the amount hyperphosphorylated tau not only by activating the key kinases but also by modulating the TOR/autophagy pathways.

**FIGURE 9:**
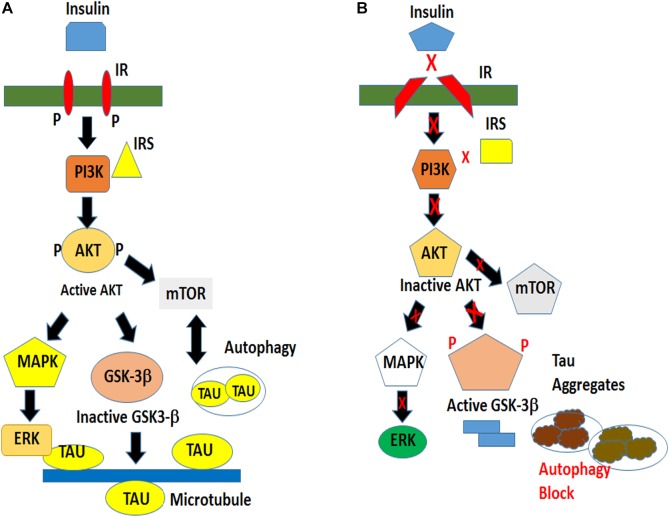
Schematic of a proposed model of tau hyperphosphorylation under the conditions of normal and impaired insulin signaling. **(A)** Insulin signaling in a physiological environment activates AKT, which in turn inactivates GSK-3β and prevents tau hyperphosphorylation. Insulin also activates TOR pathway for cellular growth and maintains a basal level of autophagy for cell survival. **(B)** An impaired insulin signaling results in an inactivation of AKT and an activation of GSK-3βY216 resulting in tau hyperphosphorylation leading to the formation of insoluble tau aggregates. The pathological condition is exacerbated by an impaired clearance of toxic protein.

Therefore, we propose a speculative mechanism of insulin sensitivity and resistance in our fly and cellular models. In an insulin-sensitive state there exists an optimal physiological level of total and phospho-tau partially controlled by the principal tau kinase GSK-3β located downstream of the insulin signaling pathway. This situation is reversed in an insulin-resistant state with dysregulation of the downstream kinases that result in the increased production of hyperphosphorylated and aggregated tau. Despite increased autophagic induction, abnormal tau accumulation is aggravated by impaired autophagic clearance ultimately causing neuronal death (Figure 9).

This study, for the first time highlights the role of Chico – the ortholog of mammalian IRSs in playing a crucial role in reducing tau hyperphosphorylation and aggregation in our tauopathy models by controlling the downstream tau kinases GSK-3β and ERK and modulating the mTOR/autophagy pathways. The recapitulation of the *in vivo* data in mammalian cells stresses the fact that the insulin signaling pathway is conserved in both systems. Our study deciphers a compelling linkage between insulin resistance in type 2 diabetes and tau pathology observed in 4R-tauopathies including AD. Further elucidation of the mechanisms may pave the way for the early detection of risk factors such as insulin resistance and designing of novel drug targets (Huang and Mucke, 2012).

## Data Availability

All datasets generated for this study are included in the manuscript and/or the Supplementary Files.

## Author Contributions

SC conducted the experiments and wrote the manuscript. SA performed the initial genetic screening with tau overexpressing *Drosophila* lines and identified Chico as a suppressor of the tau-induced “rough-eye” phenotype. AM and GJ helped in reviewing the manuscript. This study was initiated at GJ’s laboratory at the University of Texas Medical Branch, Galveston and completed at AM’s laboratory at the University of Southampton by SC as a Marie-Curie fellow.

## Conflict of Interest Statement

The authors declare that the research was conducted in the absence of any commercial or financial relationships that could be construed as a potential conflict of interest.

## Abbreviations

4EIBP: eukaryotic initiation factor 4B
AD: Alzheimer’s disease
ERK: extracellular regulating kinase
GMR: glass multimer reporter
GOF: gain of function
GSK: glycogen synthase kinase
HD: Huntington’s disease
IRS: insulin receptor substrate
LOF: loss of function
mTOR: mammalian target of rapamycin
NFT: neurofibrillary tangle
p70S6K: p70S6kinase
PD: Parkinson’s disease
T2DM: type 2 diabetes mellitus
UAS: GAL4-responsive upstream activating sequence
wt: wild type
