# Supplementary material for: Insulin-Mediated Changes in Tau Hyperphosphorylation and Autophagy in a Drosophila Model of Tauopathy and Neuroblastoma Cells
Source: Front Neurosci. 2019 Aug 2;13:801. doi: 10.3389/fnins.2019.00801 (PMC6688711; doi:10.3389/fnins.2019.00801)
Supplement: Supplementary file 1 [file Table_1.docx]

**SUPPLEMENTARY FIGURE LEGENDS**

**Figure S1. The “Rough eye” phenotype and total tau levels in Tau transgenics is not ameliorated by coexpression EGFP**. **(A)** The Tau-induced “rough eye” phenotype and the total tau levels remain unaltered when EGFP lines are coexpressed with Tau transgenics indicating a “Chico-specific” effect. **(B)** Western blot analysis from flies probed with Tau5 (Total tau) and GAPDH in the representative blots from three independent experiments. Densitometric analysis between the two genotypes shows no significant difference in the total tau levels. Genotypes: *GMR*-*gl*-Tau/+ and *GMR*-*gl*-Tau/*UAS*EGFP. (Student’s *t* test was performed for pairwise comparisons n.s= no statistical significance.

**Figure S2. Confocal images of adult retina stained with TRITC-phalloidin (red).** Ommatidial disorganization and cell loss are apparent in the eyes overexpressing the Tau transgene (B) compared with eyes expressing Controls (A), which displays a largely normal trapezoidal array of rhabdomeres. A rescue of the ommatidial loss was observed when Chico was coexpressed with Tau (C). Genotypes: *GMR*-GAL4/+; *GMR*-*gl*Tau/+; *GMR*-*g*lTau/*UAS*Chico. White arrow heads represent the rhabdomeres (membrane bound photoreceptors) in each ommatial array. Each ommatidia are magnified to show the architecture of individual photoreceptor neurons. 10 flies were used/genotype.

**Figure S3. The coexpression of Chico with Tau decreases the levels of total tau (T46) and hyperphosphorylated tau (AT8 and PHF1) while ChicoRNAi coexpression produces the opposite effect. A similar increase in the levels of total and phospho tau is observed in SY5Y cells in an insulin-resistant state. (A)** Densitometric analyses of T46, AT8 and PHF1 levels are normalized to β-tubulin. (One way ANOVA with Tukey-Kramer HSD was done for multiple group comparisons and Dunnett’s test was done for comparison with Tau, n=3 sets of Biological replicates for each genotype.). Representative blots are in Fig.2 Data represents the mean +/- SEM, ***P<0.01* and **P<0.05* relative to Tau, n.s = no statistical significance. All flies were 3 days after eclosion. Genotypes are as follows: *GMR*-GAL4/+; *GMR*-GAL4/*UAS*Chico; *GMR*-GAL4-*gl*-Tau/+; *GMR*-GAL4-*gl*-Tau/*UAS*Chico; *GMR*-GAL4/*UAS*ChicoRNAi; *GMR*-GAL4-*gl*-Tau/*UAS*ChicoRNAi. **(B)** Densitometric analyses of total tau (T46) and phosphorylated tau (AT8) normalized to GAPDH were performed based on four independent experiments. Representative blots are in Figure 5. Data represent the mean +/- S.E.M., ***P<0.01, *P<0.05* versus control (untreated cells), n=4 biological replicates. (One way Anova followed by Fisher’s test for multiple comparisons and Student’s *t* test for pairwise comparisons).

**Figure S4.** **P-mTOR protein levels are reduced in Tau transgenics and partially rescued by coexpressing Chico in Tau+Chico flies. In SY5Y cells prolonged treatment with insulin decreases the levels of P-mTOR in an insulin-resistant state.** **(A)** Western blot data shows partially rescued P-mTOR levels in Tau+Chico flies. Quantification of the levels of P-mTOR/TOR were done. (One way Anova with Tukey-Kramer HSD was done for multiple group comparisons, n=3 sets of Biological replicates for each genotype. Two-tailed student’s t-test was done for pairwise comparison of two genotypes). Data represents the mean +/- SEM, **P<0.01 relative to Tau. All flies were 3 days after eclosion. Genotypes: *GMR*-GAL4/+; *GMR*-GAL4/*UAS*Chico; *GMR*-GAL4-*gl*-Tau/+; *GMR*-GAL4-*gl*-Tau/*UAS*Chico. **(B)** Western blot data shows reduced P-mTOR levels in SY5Y cells following prolonged treatment with insulin in an “insulin-resistant” state. Densitometric analysis of these antibodies relative to GAPDH was performed based on four independent experiments. Data represent the mean +/- S.E.M, (*p<0.05) in control (untreated cells) vs. 4-hour-treated cells, n = 4 Biological replicates (One way Anova followed by Fisher’s test for multiple comparisons and Student’s t test for pairwise comparisons).

**Figure S5.** **Induction of autophagy is accompanied by** **autophagic blockage is in Tau and Tau+Chico-LOF transgenics.** Western blot data shows increased Atg8a-II/Atg8a-I ratio in Tau and Tau+ChicoRNAi flies. β-Actin was used as a loading control. (One way Anova with Tukey-Kramer HSD was done for multiple group comparisons and post-hoc Dunnett’s test was done for comparison with Tau, n=3 sets of Biological replicates for each genotype.) Data represents the mean +/- SEM, ***P<0.01* and ****P<0.001* relative to Tau. All flies were 3 days before eclosion. Genotypes: *GMR*-GAL4/+; *GMR*-GAL4/*UAS*Chico; *GMR*-GAL4/*UAS*Chico-RNAi; *GMR*-GAL4/*UAS*ChicoLOF *GMR*-GAL4-*gl*-Tau/+; *GMR*-GAL4-*gl*-Tau/*UAS*Chico; *GMR*-GAL4-*gl*-Tau/*UAS*Chico-RNAi.
