# Supplementary material for: Insulin-Mediated Changes in Tau Hyperphosphorylation and Autophagy in a Drosophila Model of Tauopathy and Neuroblastoma Cells
Source: Front Neurosci. 2019 Aug 2;13:801. doi: 10.3389/fnins.2019.00801 (PMC6688711; doi:10.3389/fnins.2019.00801)
Supplement: Supplementary file 2 [file Table_2.DOCX]

**Climbing Assay Protocol**

This assay was performed on cohorts of 15 adult flies treated with 1mM (low dose) and 10mM (high dose) Metformin. Each week, 6–9 h into the 12-hour light cycle of the flies, they were anaesthetised very briefly with CO2 and placed in a measuring cylinder in an assay room with controlled lighting conditions, temperature (23 °C) and humidity (30–40%). They were given 15 min to recover from anaesthesia and to acclimatiseto the assay room. The measuring cylinder was tapped 3 times upon amouse pad to send the flies to the bottom, a video recording was carriedout and paused 10s later when the analysis was conducted. Flies restedfor 2 min, and the procedure was repeated 2 more times. Flies were thenplaced onto fresh food until the following week.

**Survival Assay Protocol**

Three cohorts of 10 male flies of each genotype were separated 0–3 days post-eclosion and then transferred to new food twice a week and scored for deaths three times a week. Flies were housed in a room with controlled lighting conditions, temperature (23 °C) and humidity (30–40%). A Kaplan-Meier survival curve was plotted and a Log-rank (Mantel-Cox) test was performed on the data using GraphPad Prism software.
