# Supplementary figures and images for: Insulin-Mediated Changes in Tau Hyperphosphorylation and Autophagy in a Drosophila Model of Tauopathy and Neuroblastoma Cells
Source: Front Neurosci. 2019 Aug 2;13:801. doi: 10.3389/fnins.2019.00801 (PMC6688711; doi:10.3389/fnins.2019.00801)

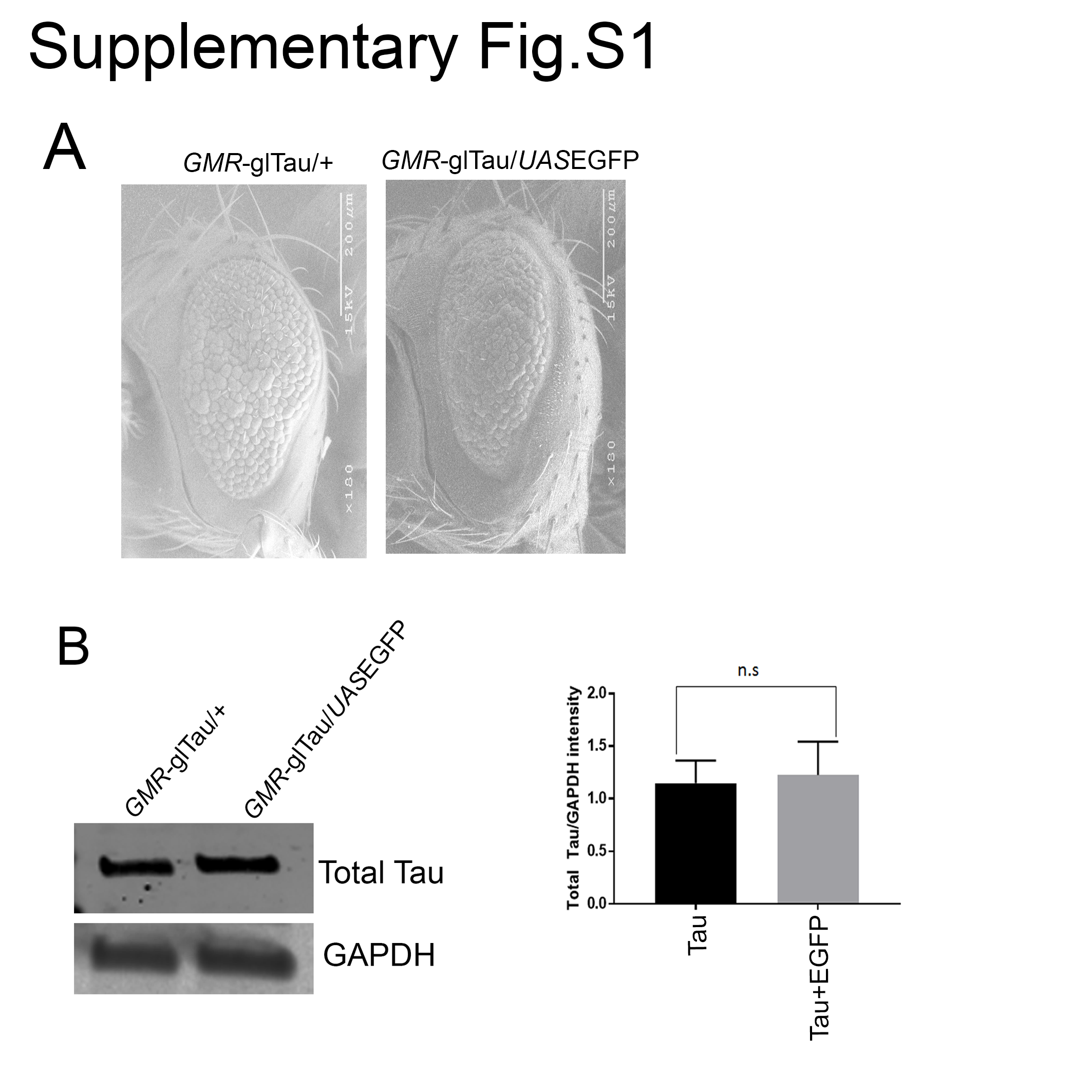

Supplement: Supplementary file 3 [file Image_1.TIF]

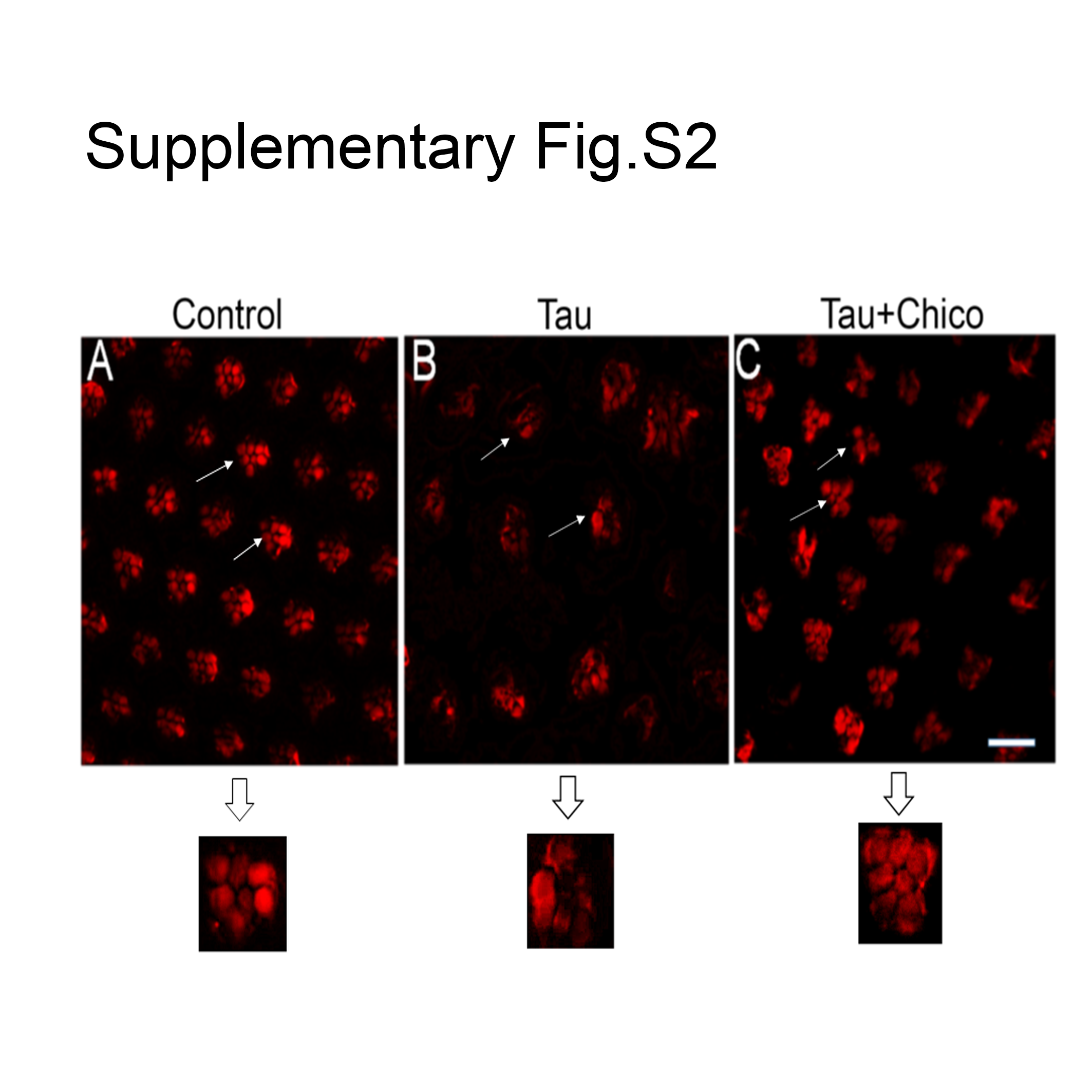

Supplement: Supplementary file 4 [file Image_2.TIF]

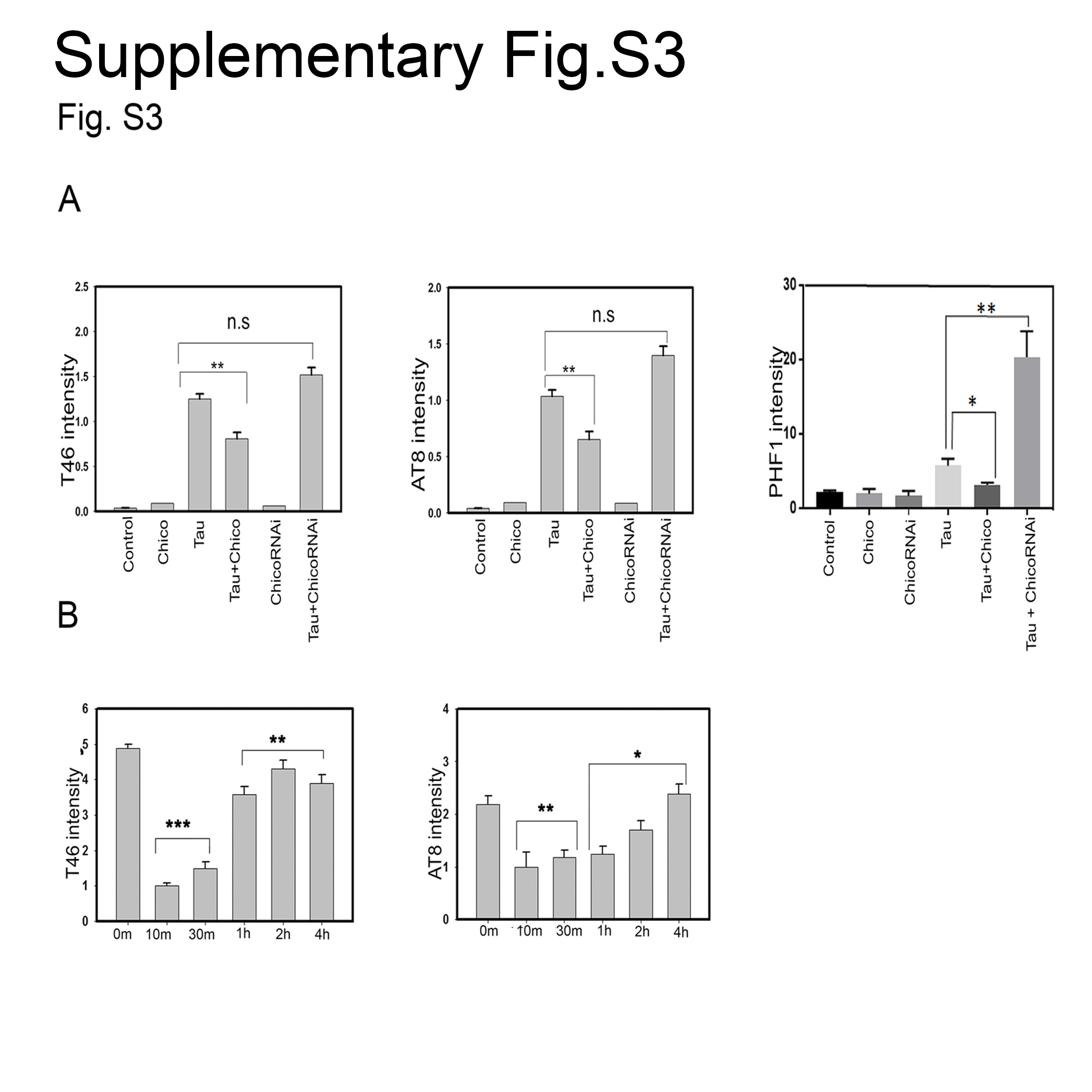

Supplement: Supplementary file 5 [file Image_3.TIF]

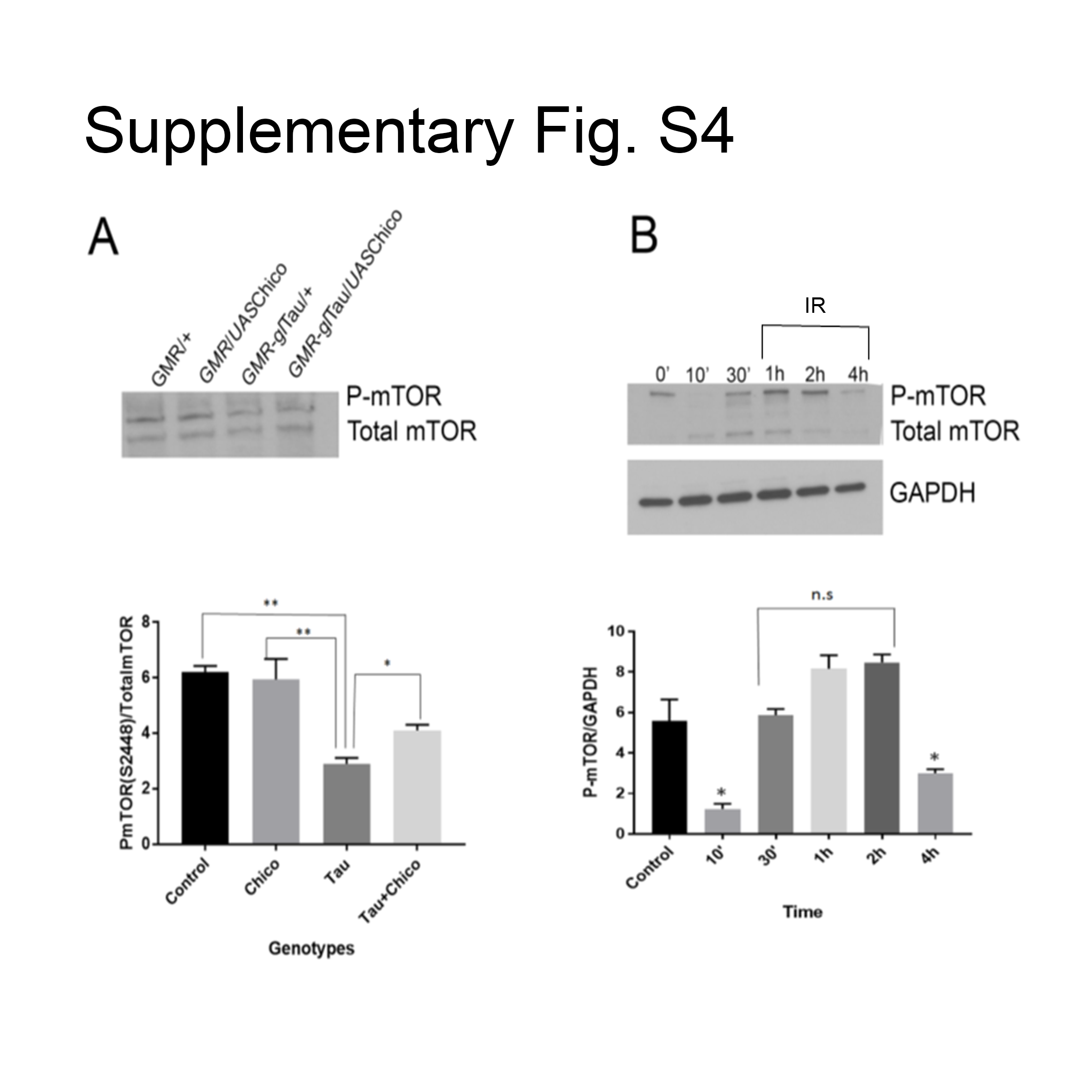

Supplement: Supplementary file 6 [file Image_4.TIF]

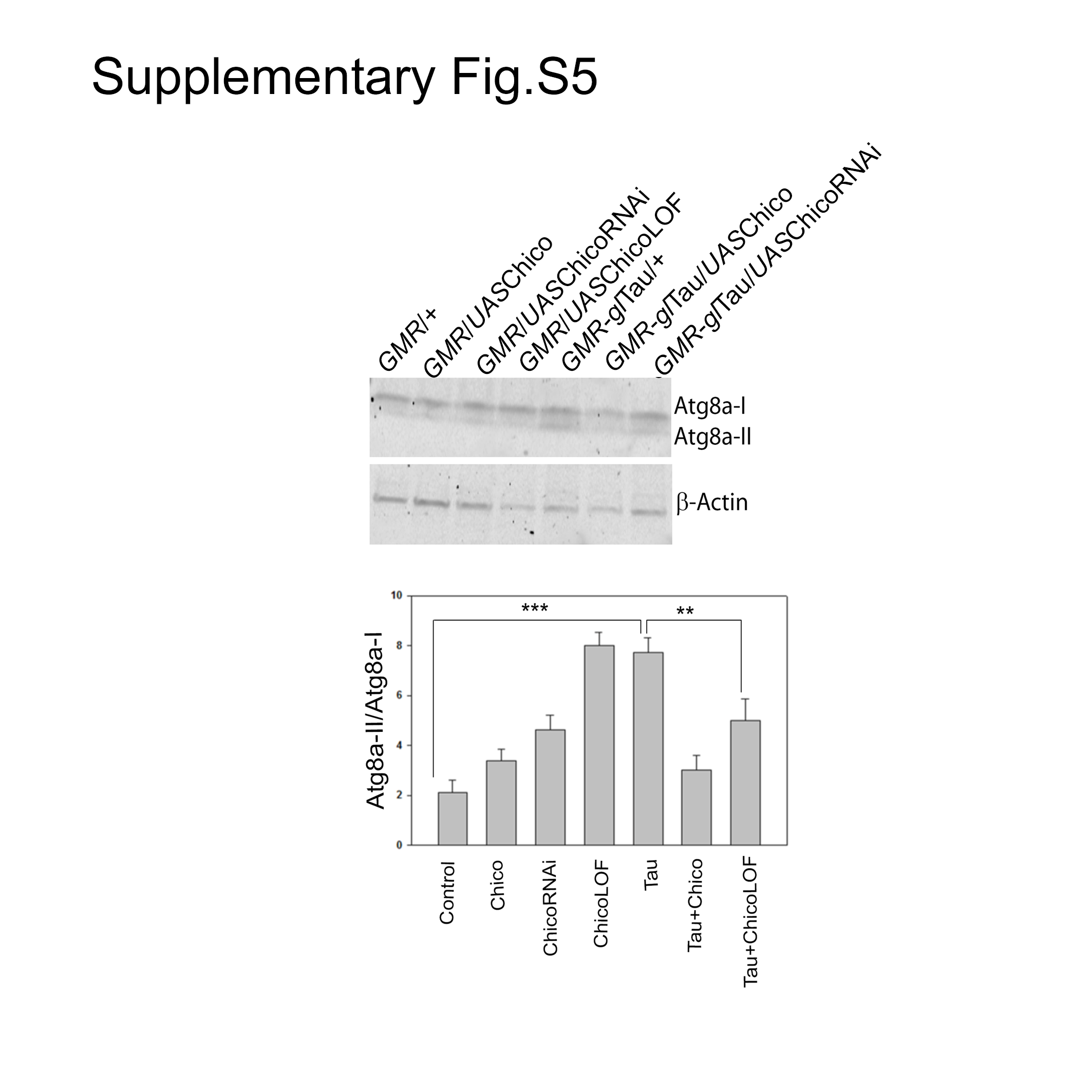

Supplement: Supplementary file 7 [file Image_5.TIF]
